# Simple periprocedural precautions to reduce Doppler microembolic signals during AF ablation

**DOI:** 10.1007/s10840-021-01010-1

**Published:** 2021-05-31

**Authors:** Marian Christoph, David Poitz, Christian Pfluecke, Mathias Forkmann, Yan Huo, Thomas Gaspar, Steffen Schoen, Karim Ibrahim, Silvio Quick, Carsten Wunderlich

**Affiliations:** 1 Technische Universität Dresden, Campus Chemnitz, Klinikum Chemnitz, Flemmingstrasse 2, 09116 Chemnitz, Germany; 2 Technische Universität Dresden, Heart Center Dresden, Fetscherstrasse 76, 01307 Dresden, Germany; 3 Klinikum Coburg, Ketschendorfer Strasse 33, 96450, Coburg, Germany; 4 Technische Universität Dresden, Klinikum Pirna, Struppener Strasse 13, 01796 Pirna, Germany

**Keywords:** Cardiac arrhythmias, Ablation, Embolic embolism, Contact force monitoring

## Abstract

**Background:**

Doppler microembolic signals (MES) occur during atrial fibrillation ablation despite of permanent flushed transseptal sheaths, frequent controls of periprocedural coagulation status and the use of irrigated ablation catheters

**Purpose:**

To investigate the number and type of MES depending on the procedure time, prespecified procedure steps, the activated clotting time (ACT) during the ablation procedure and the catheter contact force.

**Methods:**

In a prospective trial, 53 consecutive atrial fibrillation patients underwent pulmonary vein isolation by super-irrigated “point-by-point” ablation. All patients underwent a periinterventional, continuous transcranial Doppler examination (TCD) of the bilateral middle cerebral arteries during the complete ablation procedure.

**Results:**

An average of 686±226 microembolic signals were detected by permanent transcranial Doppler. Thereby, 569±208 signals were differentiated as gaseous and 117±31 as solid MES. The number of MES with regard to defined procedure steps were as follows: gaseous: [transseptal puncture, 26 ± 28; sheath flushing, 24±12; catheter change, 21±11; angiography, 101±28; mapping, 9±9; ablation, 439±192; protamine administration, 0±0]; solid: [transseptal puncture, 8±8; sheath flushing, 9±5; catheter replacement, 6±6; angiography, not measurable; mapping, 2±5; ablation, 41±22; protamine administration, 0±0]. Significantly less MES occurred with shorter procedure time, higher ACT and the use of tissue contact force monitoring.

**Conclusion:**

The current study demonstrates that during atrial fibrillation ablation using irrigated, “point-by-point” RF ablation, masses of microembolic signals are detected in transcranial ultrasound especially in the period of RF current application. The number of MES depends on the total procedure time and the reached ACT during ablation. The use of contact force monitoring might reduce MES during RF ablation.

**Supplementary Information:**

The online version contains supplementary material available at 10.1007/s10840-021-01010-1.

## Introduction

Despite of permanent flushed transseptal sheaths, frequent controls of periprocedural coagulation status and the use of irrigated ablation catheters, there is evidence that microembolic events occur during atrial fibrillation ablation [1]. Silent and only MR tomographically detectable cerebral lesions occur in 4 to 35% of cases. The effect on cognitive function remains controversial [2]. To date, it is absolutely unclear at which procedural steps during atrial fibrillation ablation the microembolic signals (MES) occur. Other questions concerning the MES are whether the total procedural time, the periprocedural activated clotting time (ACT) and the use of contact force monitoring are parameters that influence the occurrence of MES.

Therefore, in the current prospective trial, the number of MES was quantified with the help of transcranial Doppler examination (TCD) during AF ablation procedures. In addition to the quantification of MES during prespecified procedural steps, ACT values and catheter contact force, the MES were classified into solid or gaseous MES.

## Material and methods

### Study design

The current study was a prospective, single-centre registry performed in compliance with the guidelines for good clinical practice and the Declaration of Helsinki. The study was approved by the Institutional Ethical Review Board. All data were collected, managed and analysed at the Heart Centre, University of Dresden (ethics votum University of Dresden: EK 28409202).

The *primary endpoint* of this study was the number of microembolic signals (MESs), counted with continuous transcranial Doppler examination during AF ablation procedures. Thereby the numbers of MESs were evaluated during special periprocedural steps.

The *secondary endpoint* was the correlation of the number of MESs with procedural time, ACT values and tissue contact force during ablation.

### Study population and protocol

Eligible subjects were consecutive male or females >18 years of age suffering from symptomatic drug resistant atrial fibrillation (AF) requiring catheter ablation. No other inclusion criteria were necessary. Exclusion criteria were an interrupted anticoagulation for more than 12 h before the ablation procedure or a bridging of the vitamin K antagonist (VKA) with heparin. If a patient fulfilled all inclusion criteria and none of the exclusion criteria, the clinical data and intraprocedural data were analysed prospectively.

### Ablation procedure

As part of the clinical routine, all treated patients underwent a physical examination including neurological status on admission, immediately after ablation after recovery of the anaesthesia and 24 h after ablation. These results were documented in the patient records.

The ablation procedure was performed either under international normalised ratio (INR) of 2–3 if the patients were anticoagulated with VKA or under uninterrupted direct oral anticoagulant (DOAC) therapy (last intake in the morning of ablation procedure).

The AF ablation procedure was performed under sedation utilising midazolam fentanyl and propofol. One quadripolar diagnostic catheter was advanced into right ventricular apex and one decapolar catheter into the coronary sinus under fluoroscopic guidance. A single transseptal puncture was performed with a steerable intra-cardiac sheath (Agilis™, St Jude Medical) under fluoroscopy guidance. Upon left atrial access, a bolus of unfractionated heparin (100 IU/kg) was administered and repeated every 20 min to maintain an activated clotting time between 300 and 350s. The correction dose of heparin was determined by the operator. If atrial fibrillation was present during the procedure, an electrical cardioversion was performed. An electroanatomic map (EAM) including substrate mapping (voltage values higher than 0.5 mV were defined as healthy myocardium) of the left atrium and the pulmonary veins was acquired using a circular mapping catheter under fluoroscopic guidance using CARTO or NavX system. During the EAM acquisition, selective angiographies of the pulmonary veins were done for fluoroscopic confirmation of catheter position throughout PVI. Subsequently, in all atrial fibrillation procedures, a point-by-point pulmonary vein isolation was performed with pace and ablate technique with super-irrigated ablation catheters (CoolFlex (SJM) or ThermoCool Surround flow (Biosense Webster) under guidance of EAM and if necessary under active fluoroscopy (ablation settings: 40W at an irrigation rate of 15/17 ml/min with a maximum temperature of 43°C, reduction of the RF energy to 30W at the left atrial posterior wall, intra-oesophageal temperature monitoring with RF stop cut off of 39°C). During the procedure, the circular mapping catheter and the ablation catheter were changed several times via the transseptal sheath as often as necessary. Finally, the conformation of PVI and bidirectional block of all additional lines was performed under EAM and if necessary fluoroscopic guidance. At the end of the procedure, after withdrawal of the intra-cardiac sheath into the right atrium, in all patients, the heparin was antagonised with protamine (with the equal dose of heparin, maximum of 10,000 units). Finally, all sheaths were removed in the EP laboratory, the access sites were compressed manually, and a pressure bandage was applied for 6 h.

In 15 patients, the contact force during the ablation was continuously measured with the Biosense Webster Smart Touch SF catheter. The aim contact force was 5 to 40 g.

### Transcranial Doppler examination (TCD)

The continuously TCD of bilateral middle cerebral arteries during the whole AF ablation procedure was performed from a transtemporal window with a multigated Doppler-Box X system (DWL Compumedics Germany) with the help of automated MES detection and differentiation between gaseous or solid MES and artefacts. The transducers were held in place by a proprietary headpiece supplied with the system. The transducers insonate simultaneously at both 2.0 and 2.5 MHz and the system works with an automated event detection system with a previously evaluated detection and discrimination system of MESs (Supplemental Figure) [3]. During the whole ablation procedure, prespecified time markers (begin and end of transseptal puncture; begin and end of catheter change; begin and end of sheath flushing; begin and end of angiography of the pulmonary veins; begin and end of electroanatomic mapping; begin and end of ablation; administration of protamin; time marker with the measured ACT value) were manually set in the DWL software to assign the MES to the different phases of the procedure. The resulting measurement files were exported to Excel program for further processing of the raw data.

### Statistical analysis

Data were tested for normal distribution. Results of continuous variables are expressed as means ± standard deviation. Statistical analyses were done using the 2-tailed, unpaired Student’s *t* test. Level of significance was set to *p*< 0.05. Categorical variables are presented as total number with comparison using chi-square statistics and Fisher exact test. If more than 2 groups were analysed, a one-way ANOVA test was performed. Post hoc analyses have been applied using Bonferroni method. Significance level was set to *p*< 0.05.

## Results

### Study population and procedural data

In total, 53 consecutive patients were included in the current prospective analyses. The demographics and clinical baseline characteristics are shown in Table 1. In 15 patients, atrial fibrillation ablation was performed using permanent contact force monitoring. The mean age of the patients was 64 years. Sixty percent of the patients suffered from persistent atrial fibrillation. The majority of patients (60%) were treated periinterventionally with direct oral anticoagulation. Patients had a mean CHA2DS2-VASc score of 2.3. The detailed procedural data are presented in Table 2. The mean procedure time, measured from transseptal puncture to 10 min after protamine administration, was 92 min. An average of 44 min of RF energy was required for atrial fibrillation ablation. The average energy was 36 W.

**Table 1:** Baseline characteristics

Patients [*N*]	53
Gender male [*N*] (%)	27 (51)
Age [years] (SD)	64(10)
BMI (SD)	29(4)
Paroxysmal atrial fibrillation [*N*] (%)	32 (60)
Persistent atrial fibrillation [*N*] (%)	21 (40)
Qualifying risk factors	
Ejection fraction [%] (SD)	57 (8.5)
Left atrium size [mm] (SD)	43.6 (5.5)
CHA2DS2-VASc score [mean] (SD)	2.3 (1.2)
Hypertension *N* (%)	43 (81)
Hyperlipidemia *N* (%)	11 (21)
Diabetes *N* (%)	11 (21)
Coronary heart disease *N* (%)	0 (0)
VKA (INR 2–3) *N* (%)	21(40)
DOACs *N* (%)	32 (60)
Contact force monitoring [*N*]	15/53

**Table 2:** Procedural data

Procedural time^1^ [min] (SD)	92 (16)
Ablation time [min] (SD)	44 (22)
Average impedance [Ω] (SD)	183 (126)
Average power [W] (SD)	36 (3.9)
Average temperature [C°] (SD)	32.1 (1.5)

^1^Transseptal puncture - 10 min after protamine administration

In all 53 study subjects, no clinical relevant change of the neurological status could be revealed after ablation procedure, by a standardised clinical examination.

### Periprocedural number of microembolic signals (MESs) (primary endpoint)

**Fig. 1:**
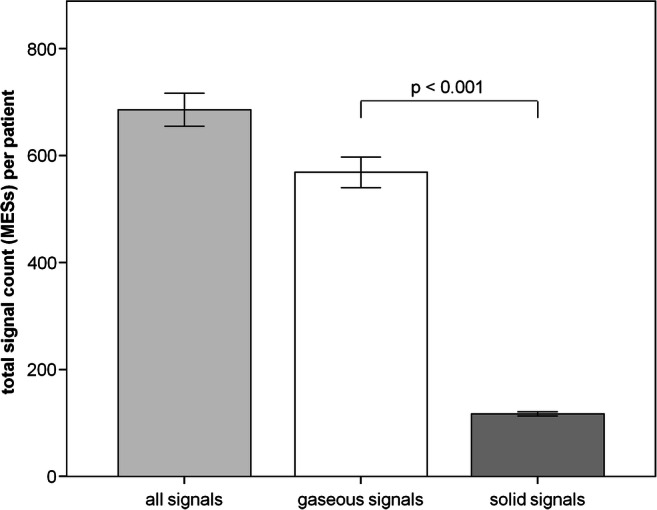
Total number of MES during AF ablation procedure. MES, microembolic signals; data presented as mean±SEM, *p*<0.05 was defined as significant

The periinterventional number of MES is illustrated in Fig. 1. During the total ablation procedure, an average of 686 ± 226 microembolic signals were detected with the help of the permanent transcranial Doppler. Of signals, 569 ± 208 were differentiated as gaseous and 117 ± 31 signals as solid.

**Fig. 2:**
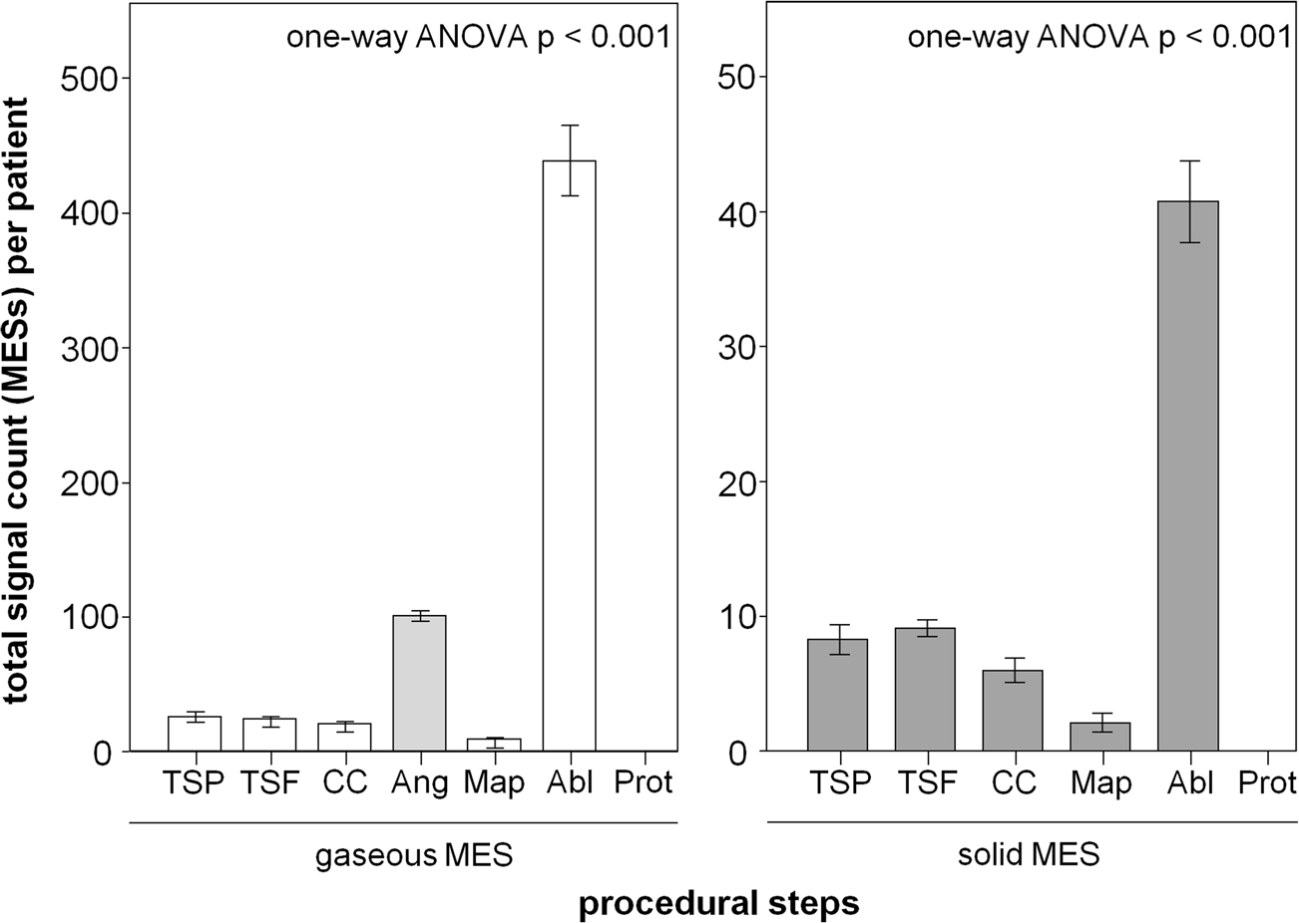
MES during the individual procedure steps of AF ablation procedure. TSP, transseptal puncture; TSF, flushing of the transseptal sheath; CC, catheter exchange through the transseptal sheath; Ang, angiography of the pulmonary veins; Map, electroanatomic map; Abl, irrigated RF ablation; Prot, 10min after administration of protamine. Data presented as mean±SEM, *p*<0.05 was defined as significant

Considering the number of microembolic signals as a function of the individual procedural steps, it is evident that the MES were not evenly distributed throughout atrial fibrillation ablation (Fig. 2). They occurred predominantly during angiography of the pulmonary veins and during RF ablation (gaseous: TSP, 26±28; TSF, 24±12; CC, 21±11; Ang, 101±28; Map, 9±9; Abl, 439±192; Prot, 0±0; solid: TSP, 8±8; TSF, 9±5; CC, 6±6; Ang, not validly measurable; Map, 2±5; Abl, 41±22; Prot, 0±0). It can be seen that significantly more gaseous than solid MES occurred at each procedural step. A mass of MES were regularly detected during angiography of the pulmonary veins, so that differentiation between gaseous and solid signals was not valid. Because the massed MES were most likely small air bubbles trapped in the radiographic contrast medium, these MES were counted as gaseous. Both the transseptal puncture itself and the exchange of the circular mapping catheter and the ablation catheter via the transseptal sheath cause only negligibly few signals compared to the total proportion of microembolic signals.

### Correlation of the number of MESs with procedural time, ACT values and tissue contact force during ablation (secondary endpoint)

**Fig. 3:**
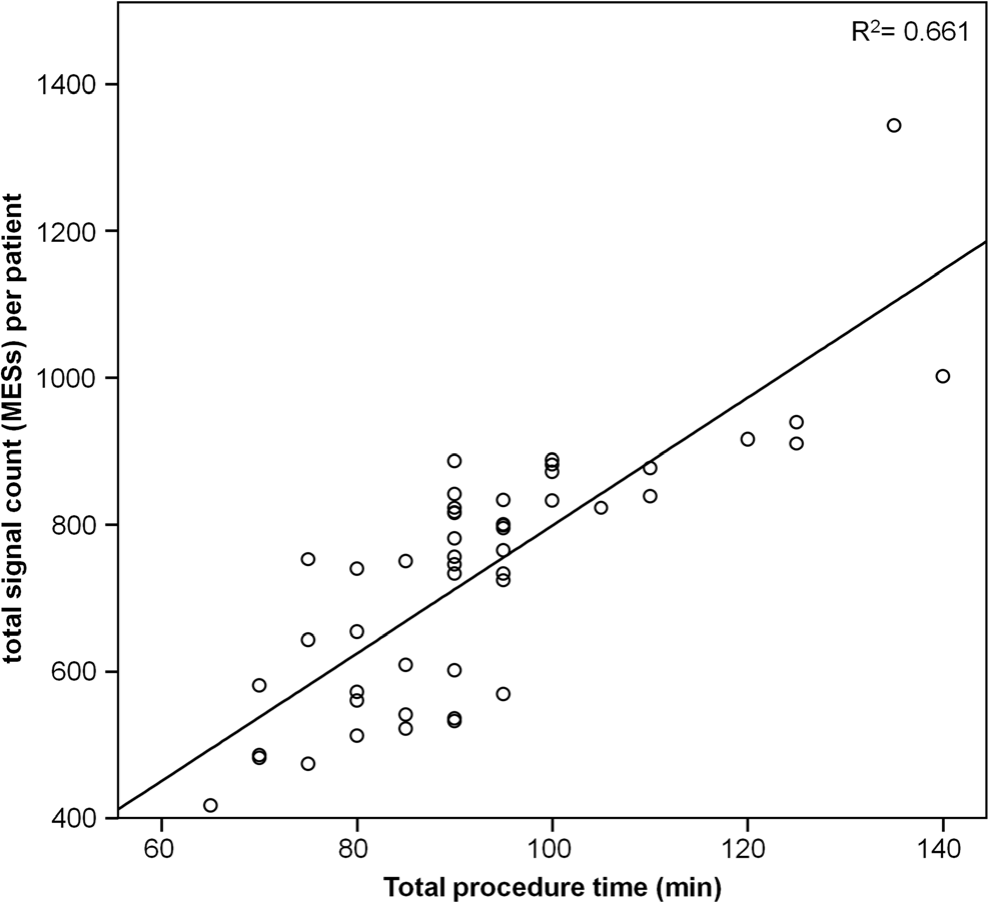
Correlation of total duration of AF ablation procedure with MESs. MES, microembolic signals; R2, Pearson correlation coefficient

Looking at the number of MESs in relation to the procedure duration (Fig. 3), there is a positive correlation counting from 417 to 1344 MESs in the registered procedures in dependence on the procedure duration (Pearson correlation coefficient of 0.661).

**Fig. 4:**
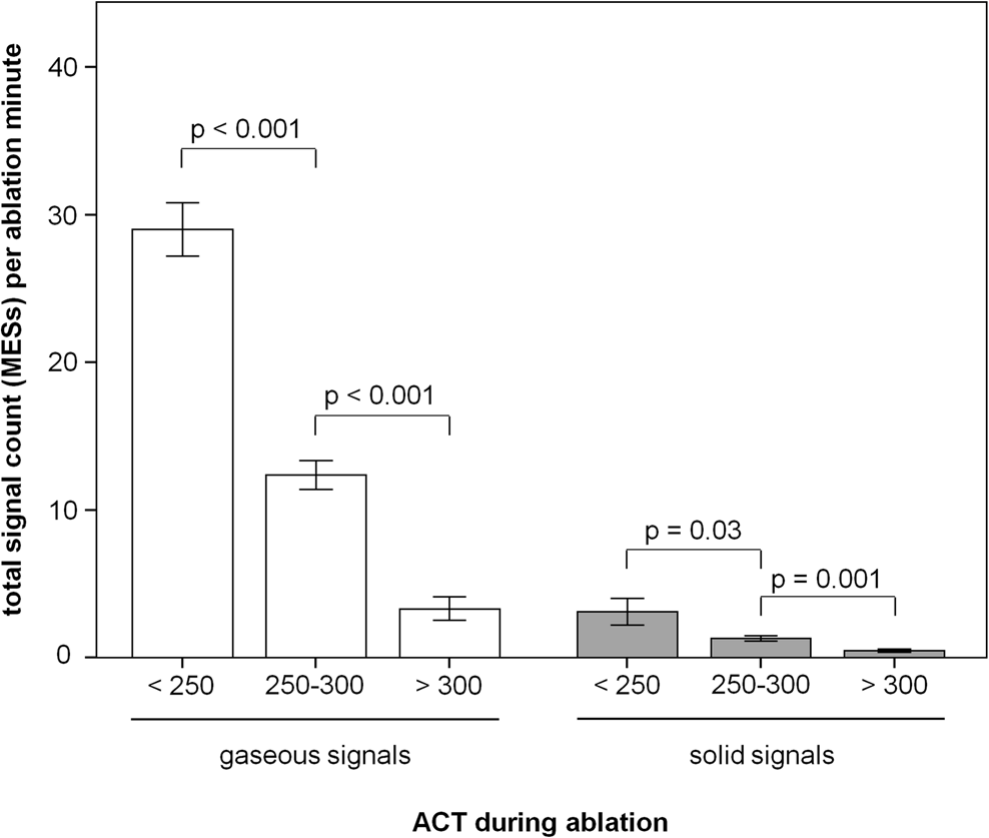
MES in dependence on the ACT. MES, microembolic signals during RF ablation; ACT, activated clotting time; data presented as mean±SEM, *p*<0.05 was defined as significant

In evaluating the number of microembolic signals as an effect of coagulation situation (activated clotting time ACT), significantly more MES were registered under low ACTs (<250 s) compared with the target ACT >300 s (Fig. 4). Thereby, 29±4 gaseous MES/ablation minute were detected at ACT < 250s; 12±3 gaseous MES/ablation minute at ACT between 250 and 300s; and only 3±3 gaseous MES/ablation minute at ACT greater than 300s (ACT<250 vs ACT 250–300 *p* < 0.001; ACT 250–300 vs ACT>300 *p* < 0.001). The situation was similar for solid signals. Here, 3±2 solid MES were counted for ACT<250s; 1±0.5 fixed MES for ACT of 250–300s; and 0.5±0.4 fixed MES for ACT greater than 300s (ACT<250 vs ACT 250-300 *p* = 0.03; ACT 250–300 vs ACT>300 *p* = 0.001).

**Fig. 5:**
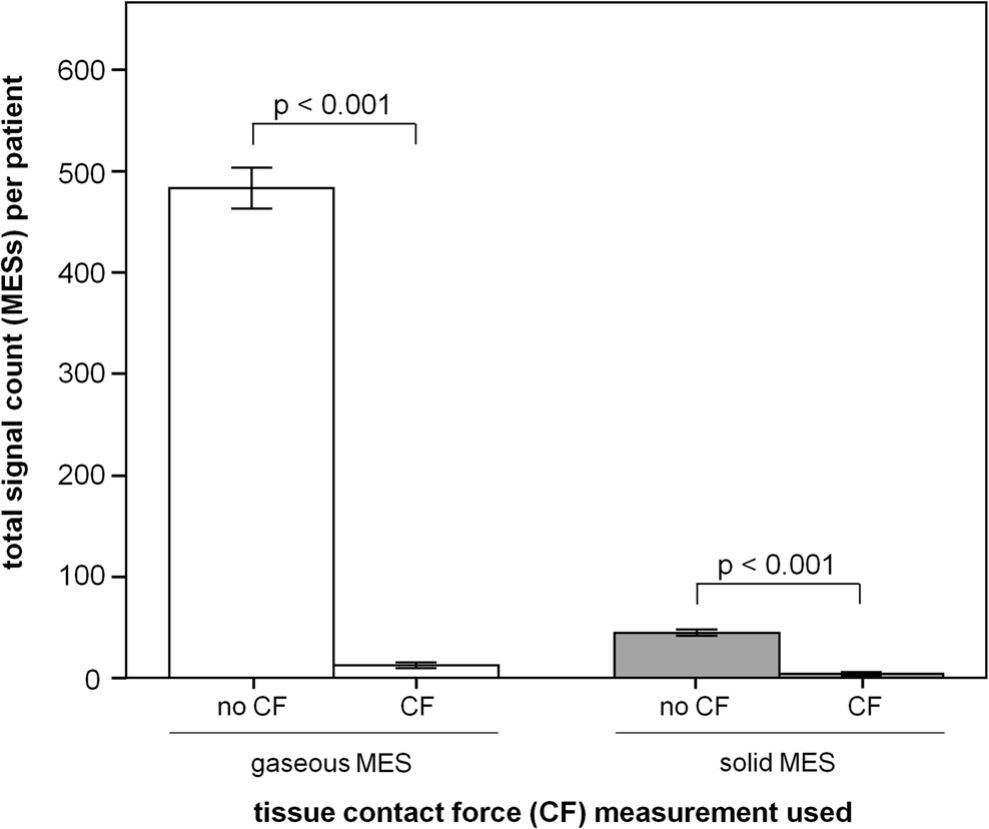
Number of MES as a function of catheter contact force monitoring. No CF, no contact force monitoring; CF, contact force monitoring; MES, microembolic signals; data presented as mean±SEM, *p*<0.05 was defined as significant

In a subset of 15 patients, atrial fibrillation ablation was performed using a catheter capable of permanent contact force monitoring (Fig. 5). A target contact force between 5 and 40g was attempted. It could be shown that under ablation without contact force measurement, significantly more gaseous MES 483±139 occurred compared to the procedures with permanent contact force monitoring 12±6 MES (no CF vs. CF *p* < 0.001). The situation was similar for solid MES. Here, an average of 45±19 MES were registered in the group without contact force monitoring compared to 4±3 MES in the contact force group (no CF vs. CF *p* < 0.001).

## Discussion

The salient finding of the present study is the observation that during atrial fibrillation ablation using irrigated, “point-by-point” RF ablation, masses of microembolic signals are detected in transcranial ultrasound. Thereby, a large part of the signals can be differentiated as gaseous signals. But also few solid signals are counted during each procedure. Thereby, during the entire procedure, the most of the MES were detected for the period of the RF current application. The present study revealed three very important factors that influence the number of MES during the AF ablation procedure: (1) the total procedure time; (2) the appropriate periprocedural anticoagulation with an achieved ACT higher than 300 s; and (3) the use of contact force monitoring with a target tissue contact force between 5 and 40 g. In this context, all these three factors can be controlled very easily in the clinical routine.

Pulmonary vein isolation using radiofrequency ablation has evolved to an effective and safe technique for the treatment of atrial fibrillation. But this procedure, similar to other procedures performed in the left circulatory system, such as transfemoral implantation of aortic valves, is suspected of causing subclinical, microembolic brain lesions [4, 5]. Furthermore, there are concerns that these microemboli will manifest themselves in the further clinical course in cognitive disorders of the patients [6].

In the current study, an average of 686 microembolic signals was counted in the transcranial Doppler during atrial fibrillation ablation. The majority (569 signals) were gaseous signals. The remaining 117 signals were classified as signals from solid emboli. In a paper by Sauren et al., microembolic signals were also detected during pulmonary vein isolation with unirrigated “point-by-point” ablation, irrigated “point-by-point” ablation and cryoballoon technology. An average of 935 microembolic signals was counted in the irrigated RF ablation group [7]. There are some reasons for this significant difference of almost 250 signals. For example, in Sauren’s study, simply irrigation catheters with only 12 irrigation holes were used. The use of these simply irrigated ablation catheters results in a significantly higher catheter tip temperature during RF ablation (approx. 48°C) compared to the super-irrigated catheters with 56 irrigation holes (approx. 30°C) used in this study. This higher catheter-tissue temperature could lead to increased gas development and thus to more embolic signals. Another study, which measured MES during AF ablation with the cryoballoon, also differentiated the MES into solid and gaseous signals as in the present work. This study shows as well that the majority of the signals are gaseous signals [8]. Another multicentre study by a German research group confirms the mass occurrence of embolic, predominantly gaseous signals during atrial fibrillation ablation [9]. In the current work, the embolic signals were counted during the following prespecified procedural steps: transseptal puncture, catheter flushing, catheter exchange via transseptal sheath, angiography of the pulmonary veins, electroanatomical mapping, irrigated RF ablation and finally during admission of protamine. It could be shown that only a few gaseous and solid microembolic signals were detectable in the procedural steps until RF current application itself. Both the transseptal puncture itself and the exchange of the mapping catheter and the ablation catheter via the transseptal sheath cause only negligibly few signals compared to the total proportion of microembolic signals. The largest amount of microembolic signals could be detected during RF ablation. This finding is confirmed in a paper by Miyazaki et al. [8]. These findings suggest that the kind of left atrial access as single or double transseptal puncture seems to be irrelevant with regard to the amount of microembolic signals. Also the number of changes between the mapping and ablation catheter via the single transseptal sheath does not matter in the context of MES. Another very interesting result of the present analysis is that after the intravenous administration of prothrombogenic protamine at the end of the ablation procedure, not a single microembolic signal is detectable until 10 min after protamine administration. The administration of protamine directly after atrial fibrillation ablation to antagonise the perioperatively administered heparin is clinically very controversial. On the one hand, protamine administration is supposed to prevent postoperative bleeding at the puncture sites. On the other hand, there is a fear that protamine administration could promote thromboembolism. However, on the basis of the current data, postprocedural protamine administration seems not to increase the MES directly after the procedure.

The current trial shows that the number of MES increases continuously with the duration of ablation.

This was an expected result that underscores that effective and sustained isolation of the pulmonary veins should be achieved in the setting of atrial fibrillation ablation without prolonging the procedure time by unnecessary or ineffective ablation points.

The procedural analyses of 15 representative cases of atrial fibrillation ablation nicely illustrate that the use of contact force monitoring enables a pronounced reduction in MES. This finding is well explained, as uncontrolled tissue contact can lead to either to increased gas development if the pressure is too low or tissue charring if the pressure is too high [10].

The current study investigated for the first time the number of microembolic signals during atrial fibrillation ablation in relation to the achieved ACT. It could be shown that patients with a higher ACT had significantly fewer microembolic signals during ablation. Thus, the number of microembolic signals was increased by a factor of 10 at an ACT < 250 s compared to an ACT > 300 s.

A recent work addressing a similar topic also demonstrated that periinterventional anticoagulation protects against microembolic signals depending on the INR value [11].

Whether a further increase of ACT higher than 400 s leads to a further decrease of thromboembolic signals without a relevant increase of the bleeding risk has not been investigated in the present work.

To interpret the current results in a valid context of clinically relevant microembolic events, it is very important to emphasise that there is no evidence that Doppler-detected microembolic signals (MES) as measured in the current work correlate with the occurrence of silent microembolic lesions on MRI. Comparing the results from previous studies that detected microembolic lesions by MRI suggests that there is no direct correlation between currently defined Doppler MES and silent MRI lesions. Whether there is a threshold for the signal intensity or type of Doppler MES that correlates with MRI lesions is absolutely unclear and remains speculative.

Since no patient in the current trial had a new neurological deficit after ablation, there is no evidence for a correlation of MES with clinical relevant neurological complications. Of note, this registry was not designed or powered to investigate the neurological outcome after AF ablation. Because a detailed assessment of the cognitive function was not performed in the current trial, the influence of MES on patient cognition remains unanswered in this study.

There are some limitations of the current study. First, the number of the included subjects is relatively small, which could influence the validity of the results and the conclusions of this study. Especially the number of patients treated with contact force monitoring was very small, so that these data should be interpreted as preliminary findings. Second, the study was not designed to investigate the clinical relevance of the MES. A statement about the correlation of microembolic signals with clinically manifest complications is not possible because of the low number of cases. Thus, the clinical relevance of MES should be investigated in larger trials with a sufficient follow-up time. Further, Markus et al. determined a sensitivity of 50.3% and a specificity of 96.5% for detecting solid embolic signals with the Doppler-Box X system, used in the current analyses [3]. Considering this diagnostic accuracy, the number of MES in the current work would be underestimated rather than overestimated. As the relatively low sensitivity of this system is a systematic error during the entire procedure, the main findings about the proportion frequency of MES during the individual procedure steps, as well as the dependence on time duration, ACT and contact force, remain unaffected. Additionally, Russell et al. describe that the specificity of the used Doppler system is predominantly dependent on the density of the signals [12]. Therefore, in the current work, no discrimination was made between solid and solid MES during the detection of MES showers during the angiographies.

## Conclusions

The current study demonstrates that during atrial fibrillation ablation using irrigated, “point-by-point” RF ablation, masses of microembolic signals are detected in transcranial ultrasound especially in the period of RF current application. The number of MES depends on the total procedure time and the reached ACT during ablation. The use of contact force monitoring might reduce MES during RF ablation. Whether Doppler MES correlate with silent microembolic lesions on MRI remains unclear.

## Supplementary Information

ESM 1 Methods. TSP, transseptal puncture; TSF, flushing of transseptal sheath; CC, catheter change; Ang, angiography of the pulmonary veins; Map, electroanatomical mapping; Abl, RF current application; Prot, time period of 10 min after administration of protamine (PNG 10460 kb) (TIF 2843 kb)

High Resolution Image (TIF 2843 kb) (PNG 10460 kb)

BMI, body mass index; VKA, vitamin K antagonist; INR, international normalised ratio; DOACs, direct oral anticoagulant

## Data Availability

The data that support the findings of this study are available from the corresponding author, MC, upon reasonable request.
