# Supplementary figures and images for: Simple periprocedural precautions to reduce Doppler microembolic signals during AF ablation
Source: J Interv Card Electrophysiol. 2021 May 31;64(2):359–65. doi: 10.1007/s10840-021-01010-1 (PMC9399063; doi:10.1007/s10840-021-01010-1)

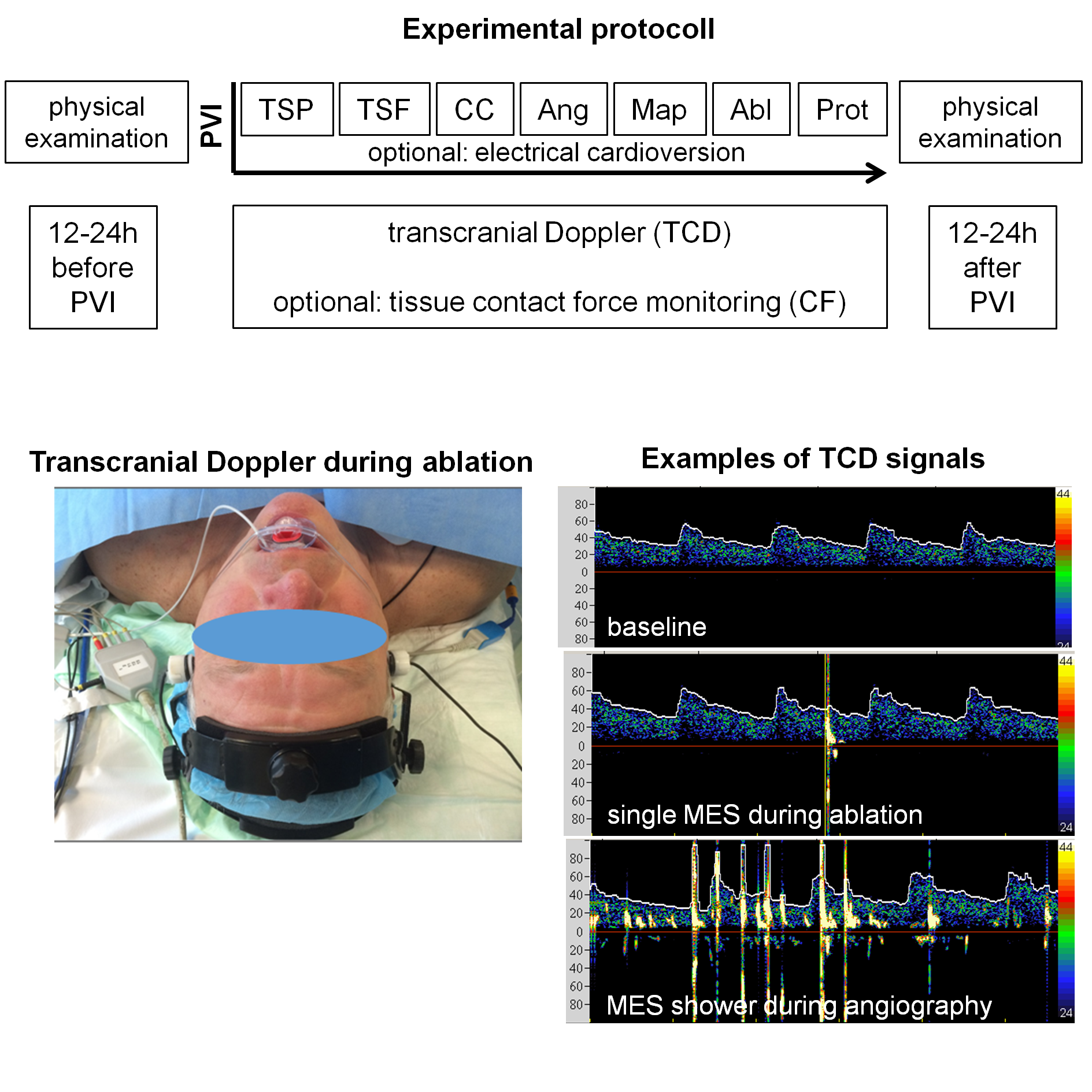

Supplement: Supplementary file 1 — Methods. TSP, transseptal puncture; TSF, flushing of transseptal sheath; CC, catheter change; Ang, angiography of the pulmonary veins; Map, electroanatomical mapping; Abl, RF current application; Prot, time period of 10 min after administration of protamine (PNG 10460 kb) (TIF 2843 kb) [file 10840_2021_1010_MOESM1_ESM.tif]

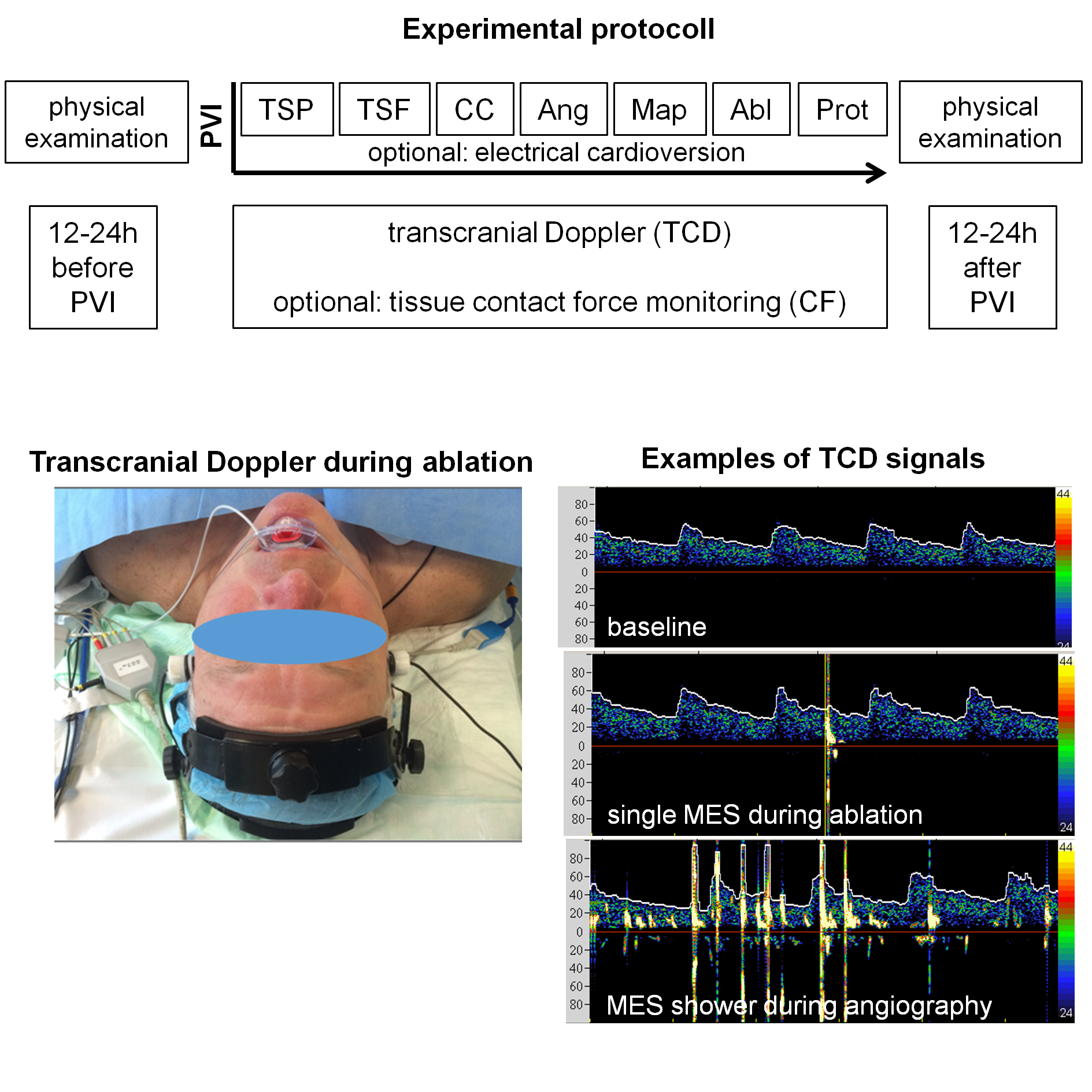

Supplement: Supplementary file 2 — High Resolution Image (TIF 2843 kb) (PNG 10460 kb) [file 10840_2021_1010_FIG6_ESM.png]
